# Development and Validation of a Fall Questionnaire for Patients with Parkinson's Disease

**DOI:** 10.1002/mdc3.13515

**Published:** 2022-07-23

**Authors:** Anika Frank, Jonas Bendig, Sophia Finkbeiner, Tom Hähnel, Nils Schnalke, Tim Feige, Heinz Reichmann, Björn H. Falkenburger

**Affiliations:** ^1^ Department of Neurology University Hospital and Faculty of Medicine Carl Gustav Carus Dresden Germany; ^2^ German Center for Neurodegenerative Diseases Dresden Germany

**Keywords:** Parkinson's disease, surveys and questionnaires, falls, validation study, fall‐related injuries

## Abstract

**Background:**

In Parkinson's disease, postural instability and falls are of particular socioeconomic relevance. Although effective fall prevention and the prophylaxis of fall‐related injuries depend on low‐threshold symptom monitoring, validated instruments are lacking.

**Objectives:**

To develop a self‐report questionnaire for the assessment of falls, near falls, fear of falling, fall‐related injuries, and causes of falls for patients with Parkinson's disease (PwPD).

**Methods:**

A pool of potential items was generated from a literature review and by discussion in an expert panel. The first version of the Dresden Fall Questionnaire (DREFAQ) was tested in a group of German‐speaking movement disorder specialists as well as PwPD. The resulting 5‐item questionnaire was assessed in a validation cohort of 36 PwPD who documented fall events and near‐fall events in a calendar for 3 months and completed the DREFAQ at the end of the study. The questionnaire was subsequently used in a separate cohort of 46 PwPD to determine test–retest reliability and confirm the factor structure.

**Results:**

The DREFAQ showed good internal consistency (Cronbach's α = 0.84) and good test–retest reliability (intraclass correlation coefficient, 0.76; 95% confidence interval, 0.60–0.86). The total DREFAQ score showed good concurrent validity with fall events (Spearman's ρ = 0.82) and near‐fall events (Spearman's ρ = 0.78) as determined by fall and near‐fall diaries. Factor analysis revealed a 2‐factor structure composed of near falls with fear of falling and severe falls with injuries.

**Conclusions:**

The DREFAQ is a reliable and valid 5‐item questionnaire for determining the incidence of falls, near falls, fear of falling, fall‐related injuries, and causes of falls in PwPD.

Unlike other neurodegenerative diseases, Parkinson's disease (PD) can be treated effectively. Nevertheless, in the late phase of the disease, symptoms become increasingly resistant to dopaminergic therapy, with postural instability and falls being of particular relevance. Up to 70% of patients with PD (PwPD) fall each year,^1^ with a large proportion (50%–86%) falling recurrently.^2^ The enormous health and socioeconomic importance of these complications is highlighted by the adverse impact on quality of life, social participation, physical functioning, and health‐related costs.^3^, ^4^, ^5^ Indeed, the costs of fall‐related fractures in PwPD are almost twice as high as those in healthy older people,^6^ and the incidence of hip fractures is 4 times that for people of the same age without PD.^7^, ^8^

The stochastic nature of falls hampers their assessment in outpatient visits or even in longer stays in the hospital. Yet, the quantification of falls is critical to assess therapeutic changes for their potential to increase or decrease falls. A structured analysis of falls might also contribute to the identification of particularly vulnerable patients who would benefit from the prescription of evidence‐based treatments. Assessing falls is therefore important for clinical routine and for studies aimed to reduce falls and their socioeconomic burden. Furthermore, because recurrent falls represent 1 of the 4 milestones in the PD milestone concept, their reduction could represent an outcome for neuroprotective strategies.^9^, ^10^

Despite the relevance of falls in the context of PD and in the geriatric population in general, there are few studies dealing with a structured determination of fall frequency. Fall calendars or fall diaries are considered the gold standard for an exact recording of fall frequency.^11^ However, these can only be used prospectively and are connected with a high level of effort for patients and caregivers.^12^ On the other hand, standardized, examiner‐based scoring systems (eg, Unified Parkinson's Disease Rating Scale [UPDRS] or the Berg Balance Scale) only allow for an approximation of the fall risk of patients. These scales, however, do not document actual fall events or their consequences and bind a high amount of expert labor. Moreover, a recent meta‐analysis and systematic review found that no fall risk assessment tool predicts elderly fallers with sufficient accuracy.^13^

In PD, several risk factors increase the risk of falling, including freezing of gait (FOG), previous falls, lower limb weakness, and cognitive impairments.^2^ Furthermore, falls occurring under certain circumstances may provide insight into underlying causes and fall prevention strategies. Falls as a result FOG and impaired balance are, for example, more frequent in patients with the postural instability and gait disorder (PIGD) subtype compared with tremor‐dominant PD.^14^ Another important aspect related to falls is fear of falling, which has been shown to be associated with a lower quality of life in a recent comprehensive systematic review.^15^

The development of a validated and reliable self‐report questionnaire for a cost‐effective detection of falls, fear of falling, and causes of falls in PD could therefore be beneficial to clinical care as well as the scientific community. The importance of examining reliability and validity in the context of fall assessment is further underlined by the unclear effects of interval‐dependent recall bias.^16^, ^17^ With the development of the Dresden Fall Questionnaire (DREFAQ), we sought to create the first PD‐specific fall assessment instrument with established reliability and validity.

## Methods

The development and validation of the questionnaire were carried out in in the following 5 successive steps: (1) item generation, (2) cognitive pretesting, (3) testing the questionnaire in the validation cohort, (4) testing in the confirmation cohort, and (5) statistical analysis and finalization.

### Development of the Questionnaire

A pool of items was generated from a literature review of known fall risks and fall‐related injuries in PD and by discussion in an expert panel.^3^, ^18^ The questionnaire was designed to cover the domains of (1) frequency of falls, (2) near falls, (3) fear of falling, (4) causes of falling, and (5) fall‐related injuries. The frequency of falls, near falls, and situations with fear of falling were evaluated quantitatively through a 4‐point ordinal scale. To stress the importance of fall‐related injuries and the circumstances in which falls occur, specific qualitative questions were added. This information was sought to provide insights into the resulting injuries to estimate the severity of falls as well as the underlying causes and fall prevention strategies.

Falls were defined as unexpected events that resulted in the participant unintentionally coming to the ground, floor, or other lower level. Near falls were defined as events in which a fall is avoided by any posture‐stabilization measures (eg, big steps, arm support, or holding onto something). The expert panel selected items based on clinical importance, relevance to PD, and importance from a patient's perspective from a larger item pool.

### Cognitive Pretesting

To assess the DREFAQ with examiners and respondents, we conducted a qualitative cognitive pretest using testing guides according to the guidelines of the International Parkinson and Movement Disorder Society (MDS) Taskforce for questionnaire design.^19^ The guide was based on qualitative techniques, verbal probing, and “think‐aloud” interviewing to identify problems with the scale from the patient and rater perspectives. German‐speaking PwPD (n = 10) and movement disorders specialists (n = 7) completed the pretesting. Scale revisions based on cognitive pretesting included changes in phrasing and simplification of questions. Two questions were merged into a single question, and 2 questions were removed from the instrument. Open questions were changed to multiple‐choice questions with a free‐text option for “others.” Response categories for items 1, 2, and 3 were changed from 0 to 4 to 0 to 3 based on results from the validation cohort.

The final version of the DREFAQ can be found in the supplementary information in the original validated German version and an unvalidated English version. Briefly, the questionnaire contains an introduction defining falls and near falls, which is followed by the following 5 questions referring to the previous 3 months: (1) “How often did you fall?”, (2) “How often did you encounter situations where you almost fell but were able to catch yourself (near falls)?”, (3) “How often did you have concerns or fear of falling?”, (4) “Have you been injured in falls? If you have been injured, please specify the type and location of your injury(ies)”, (5) “If you have fallen, please specify the circumstances.” Items 1 to 4 are scored from 0 to 3. The total score of the DREFAQ is calculated by summing the first 4 items (0–12). The location of the injury (from item 4) and the circumstances (item 5) are not used in the score calculation but provide additional qualitative information.

### Validation Cohort (n = 36)

Written informed consent was obtained from all participants before inclusion. All procedures were performed following relevant guidelines and regulations. The study was approved by the institutional review board of the Technische Universität Dresden (BO‐EK‐14012021). Patients were recruited between April and October 2021 in the outpatient clinics for movement disorders at the University Hospital Carl Gustav Carus Dresden. Inclusion criteria were the clinically probable diagnosis of idiopathic PD by a specialist in movement disorders according to the diagnostic criteria of the MDS as well as sufficient German‐language skills.^20^ Exclusion criteria were advanced dementia and inability to walk (wheelchair‐bound or bedridden).

Patients completed a baseline visit (60 minutes), followed by an independent use of a fall and near‐fall diary at home for 3 months. All participants who reported a fall were instructed to document further information in a report form to verify the circumstances of the falls and any related injuries. After 3 months, the DREFAQ was answered by the participants. The fall and near‐fall diary and the report forms were used as a gold standard for the DREFAQ. In the baseline visit, clinical and demographic data were transferred from patients' records (age, disease duration, Hoehn & Yahr stage,^21^ the presence of disease‐related complications, medication and calculated levodopa equivalent daily dose,^22^ MDS‐Sponsored Revision of the UPDRS [MDS‐UPDRS] Part III,^23^ and Beck Depression Inventory–II [BDI‐II]^24^). To evaluate further contributors for falls, the following domains were assessed with rater‐based scales or self‐report questionnaires: cognitive impairment (Functional Activities Questionnaire [FAQ],^25^ Montreal Cognitive Assessment [MoCA]^26^), orthostatic dysregulation, urge incontinence (Scales for Outcomes in Parkinson's Disease–Autonomic Dysfunction items 8–16, urinary urgency and orthostatic dysfunction categories),^27^ anxiety (Parkinson Anxiety Scale [PAS]),^28^ motor recklessness (Barratt Impulsiveness Scale–11 [BIS‐11]),^29^ FOG (Freezing of Gait Questionnaire [FOG‐Q]),^30^ activities of daily living (MDS‐UPDRS Part II),^23^ fear of falling (Falls Efficacy Scale–International),^31^ quality of life (Parkinson's Disease Questionnaire–8 [PDQ‐8]),^32^ disease severity (Patient Global Impression of Severity [PGI‐S]^33^), and personality (Big Five Inventory–10).^34^

### Confirmation Cohort (Parkinson Network Eastern Saxony [PANOS], n = 46)

Written informed consent was obtained from all participants before inclusion. All procedures were performed following relevant guidelines and regulations. The study was approved by the institutional review board of the Technische Universität Dresden (BO‐EK‐517112020). Patients were recruited between April and November 2021 at the University Hospital Dresden. The ongoing PANOS study investigates the effects of an integrated care network for PwPD on their quality of life. As a part of the network, a monitoring package with self‐report questionnaires (including the DREFAQ) was completed by the patients every 3 months. Data from patients who had both a complete baseline and the 3‐month monitoring package (February 2022, n = 46) with no missing values in the DREFAQ were included in the test–retest reliability analysis and the confirmatory factor analysis (CFA).

### Statistical Analyses

#### Sample Size

The sample size of the validation cohort was based on a calculation of 5 to 10 patients per item.^35^ The quantitative part of the DREFAQ contains 4 items; therefore, 40 patients were enrolled in the validation cohort. For the reliability analysis in the confirmation cohort, no sample size calculation was conducted. All patients who were enrolled in the study and had 2 valid measurements (baseline and 3‐month follow‐up, February 2022) of the DREFAQ were included in the reliability analysis and the CFA.

#### Factor Analysis

Exploratory factor analysis (EFA) was used in the validation data set to determine the factor structure. Kaiser–Meyer–Olkin Measure of Sampling Adequacy and Bartlett's Test of Sphericity were used with thresholds of >0.5 and <0.05 to assess data adequacy. Factors were retained based on Velicer's minimum average partial (MAP) test and parallel analysis. The threshold level for factor loading was >0.40 and for dual loading was <0.40.^36^

A CFA was performed in the confirmation cohort. To assess model fit, χ^2^ statistic, comparative fit index (CFI), Tucker–Lewis Index (TLI), Akaike information criterion (AIC), root mean square error of approximation (RMSEA), and standardized root mean square residual (SRMR) were used. The following thresholds were applied: χ^2^ <5.0, CFI >0.95, TLI >0.95, RMSEA <0.06 (*P* < 0.05), and SRMS <0.08, as suggested by others.^37^

For the 1‐factor and 2‐factor models, we obtained the following values: CFI, 0.873 and 0.996; TLI, 0.619 and 0.977; χ^2^ test, 13.428 and 1.348; AIC, 457.08 and 447.00; RMSEA, 0.352 and 0.087; and SRMR, 0.075 and 0.015. Although RMSEA was above the recommended thresholds, the model fit can still be regarded as adequate due to the small sample size and the low number of items allowing limited degrees of freedom.^38^

#### Reliability

Internal consistency was measured in the validation cohort using Cronbach's α and item‐to‐total correlations. Acceptable scores were defined as ≥0.7 for Cronbach's α and ≥0.3 for the item‐to‐total correlations.^39^, ^40^, ^41^ The test–retest reliability was assessed in the confirmation cohort (3‐month interval) using intraclass correlation coefficients (ICCs; 2‐way mixed effects, absolute agreement, single rater/measurement), with an ICC ≥0.75 indicating good reliability.^42^

#### Validity

Concurrent validity was computed by correlating/comparing the DREFAQ score and single items with information from the fall diaries in the validation cohort. Furthermore, DREFAQ scores were correlated with established health measures. Linear models were fitted to identify variables associated with the number of falls and the severity of injuries. A maximum of 4 independent variables were chosen by sequential replacement.^43^

#### Statistical Reporting and Software

Unless otherwise stated, the data are reported as mean with standard deviation. Correlation analysis, tests for significance, sensitivity/specificity analysis, tests for internal consistency, and EFA were performed using the Python programming language implemented in Jupyter notebook (packages: scipy_1.6.2, pingouin_0.3.12, factoranalyzer_0.3.2). CFA was performed using the Rstudio programming environment (R Foundation for Statistical Computing, Vienna, Austria; packages: psych_2.1.9, lavaan_0.6–10, paran_1.5.2). Analyses of ICC were performed with SPSS version 28 (IBM Corp., Armonk, NY).

## Results

### Clinical Data

A total of 40 patients were recruited for the validation cohort and completed the baseline assessment. The questionnaire and the fall diaries were returned by 36/40 patients. Two patients did not return the questionnaire, and 2 patients did not return the fall diary. These 4 patients were excluded from the study. Demographic and clinical data of the cohort are summarized in Table 1 (“validation cohort”).

**TABLE 1:** Demographic and clinical data

Clinical Variables	Validation Cohort	Confirmation Cohort	*P* Value
Subjects, n	36	46	
Age, y, mean ± SD	65.5 ± 11.5	71.6 ± 7.4	**0.0048**
Sex, female:male (% male)	7:29 (80.6%)	19:27 (58.7%)	0.0548
H&Y *on*, n	Mild (H&Y 0–2): 17 Moderate (H&Y 2.5, 3): 16 Severe (H&Y 4, 5): 3	Mild (H&Y 0–2): 6 Moderate (H&Y 2.5, 3): 24 Severe (H&Y 4, 5): 18	**0.0001**
Disease duration, y, mean ± SD	11.6 ± 7.7	8.1 ± 5.4	**0.0182**
Subtype, n	Tremor dominant: 4 Akinetic‐rigid: 16 Mixed: 16	Tremor dominant: 7 Akinetic‐rigid: 21 Mixed: 18	0.8254
LEDD, mean ± SD	763.69 ± 419.81	728.61 ± 304.05	0.6620
MDS‐UPDRS Part III, mean ± SD	15.3 ± 8.3	34.7 ± 16.2	**<0.0001**
PIGD score, mean ± SD	0.82 ± 0.63	–	
FES‐I score, mean ± SD	30.7 ± 12.51	34.89 ± 13.13	0.1508
FOG‐Q score, mean ± SD	7.65 ± 6.17	11.37 ± 6.02	**0.0072**
MoCA score, mean ± SD	25.3 ± 3.3	22.6 ± 5.2	**0.0067**
DBS, yes:no (% yes)	14:22 (38.9%)	1:45 (2.2%)	**<0.0001**

The table depicts data from the validation and confirmation cohort as mean with SD or absolute numbers per category. *P* values were calculated with independent *t*‐tests (age, disease duration, UPDRS III and MoCA), Mann–Whitney *U* test (H&Y *on*), or Fisher's exact test (sex, subtype, and DBS). Significant differences between groups are highlighted in bold.

Abbreviations: SD, standard deviation; H&Y, Hoehn & Yahr; LEDD, levodopa equivalent daily dose; PIGD, postural instability and gait disorder; MDS‐UPDRS, Movement Disorder Society–Sponsored Revision of the Unified Parkinson's Disease Rating Scale; FES‐I, Falls Efficacy Scale–International; FOG‐Q, Freezing of Gait Questionnaire; MoCA, Montreal Cognitive Assessment; DBS, deep brain stimulation.

Every second patient (18/36) fell at least once during the 3 months of the study; recurrent falls, defined by at least 1 fall per month, occurred in 6/36 patients. A slightly higher number (20/36) reported at least 1 near fall in the previous 3 months, but recurrent near fallers (at least 1 near fall per month) were more frequent (12/36). The number of near falls was slightly but significantly higher than the number of falls (median = 1 vs. median = 0.5; *P* = 0.0144, Wilcoxon test). PwPD who experienced falls were mainly injured lightly (n = 16) or not at all (n = 5). However, 2 patients reported a laceration or cut injury, and 1 patient reported a fracture. The main location for injuries was the lower extremities (n = 14) followed by injuries of the hand, arm, or shoulder (n = 9). The circumstances in which falls occurred were mainly attributed to loss of balance (n = 14) or stumbling (n = 8).

Interestingly, fear of falling (item 3) showed only a weak correlation with fall‐related injuries (Spearman's ρ = 0.32, nonsignificant). Accordingly, 9 of the 18 patients who reported at least 1 fall did not report any fear of falling (DREFAQ item 3 = 0). A detailed listing of patients' answers to the DREFAQ in the validation cohort can be found in Table S1 (validation cohort).

Patients in the confirmation cohort were significantly older, had shorter disease durations, higher Hoehn & Yahr stages, higher MDS‐UPDRS Part III ratings, and lower MoCA scores compared with the validation cohort. Treatment with deep brain stimulation was less frequent in the confirmation cohort (Table 1). Despite these differences, similar patterns of fall frequency and near‐fall frequency were observed (Table S1; confirmation cohort at baseline). Patients in the confirmation cohort reported fear of falling more often than patients in the validation cohort (*P* < 0.0001, Mann–Whitney *U* test). Moreover, every patient who experienced falls in the confirmation cohort reported fear of falling (DREFAQ item 3 >0). Types and locations of injuries as well as causes of falls were not significantly different (Table S1).

### Psychometric Properties

#### Item Analysis

In the validation cohort, responses were screened for low frequency and considered for removal. Because there were frequencies lower than 10% for the fourth response (“…1–6 times per week”) and the fifth response (“…at least one time per day”) for falls, near falls, and fear of falling, we aggregated the 2 highest responses to a singular option (“…at least once per week”).

#### Factor Structure

In the validation cohort, Velicer's MAP test suggested a 1‐factor solution, whereas parallel analysis suggested a 2‐factor solution. CFA in the confirmation cohort showed a better fit for the 2‐factor model; factor loadings are reported in Table 2. The factors account for 65.5% of the variance (factor 1, 35.0%; factor 2, 29.7%) and can be interpreted as “near falls with fear of falling” and “severe falls with injuries.”

**TABLE 2:** Factor loadings from exploratory factor analysis

Items	Factor 1, Near Falls with Fear of Falling	Factor 2, Severe Falls with Injuries
Fear of falling, item 3	**0.82**	−0.09
Near‐fall frequency, item 2	**0.69**	0.25
Severity of injuries, item 4	−0.06	**0.91**
Fall frequency, item 1	0.21	**0.71**

Extraction method: Maximum likelihood; rotation method: Oblimin. Loadings larger than 0.4 are in bold.

#### Reliability

The DREFAQ showed an acceptable internal consistency of ≥0.7 (Cronbach's α = 0.84; 95% confidence interval, 0.73–0.91) in the validation cohort. Cronbach's α could not be improved by deleting items. Corrected item‐to‐total correlations (Spearman's ρ) were above the threshold of 0.3 for all quantitative items (item 1, 0.74; item 2, 0.77; item 3, 0.58; item 4, 0.65). The ICC (0.76; 95% confidence interval, 0.60–0.86) was above the threshold of >0.75, indicating good test–retest reliability of the DREFAQ over a 3‐month interval in the confirmation cohort.

#### Construct Validity

**FIG. 1:**
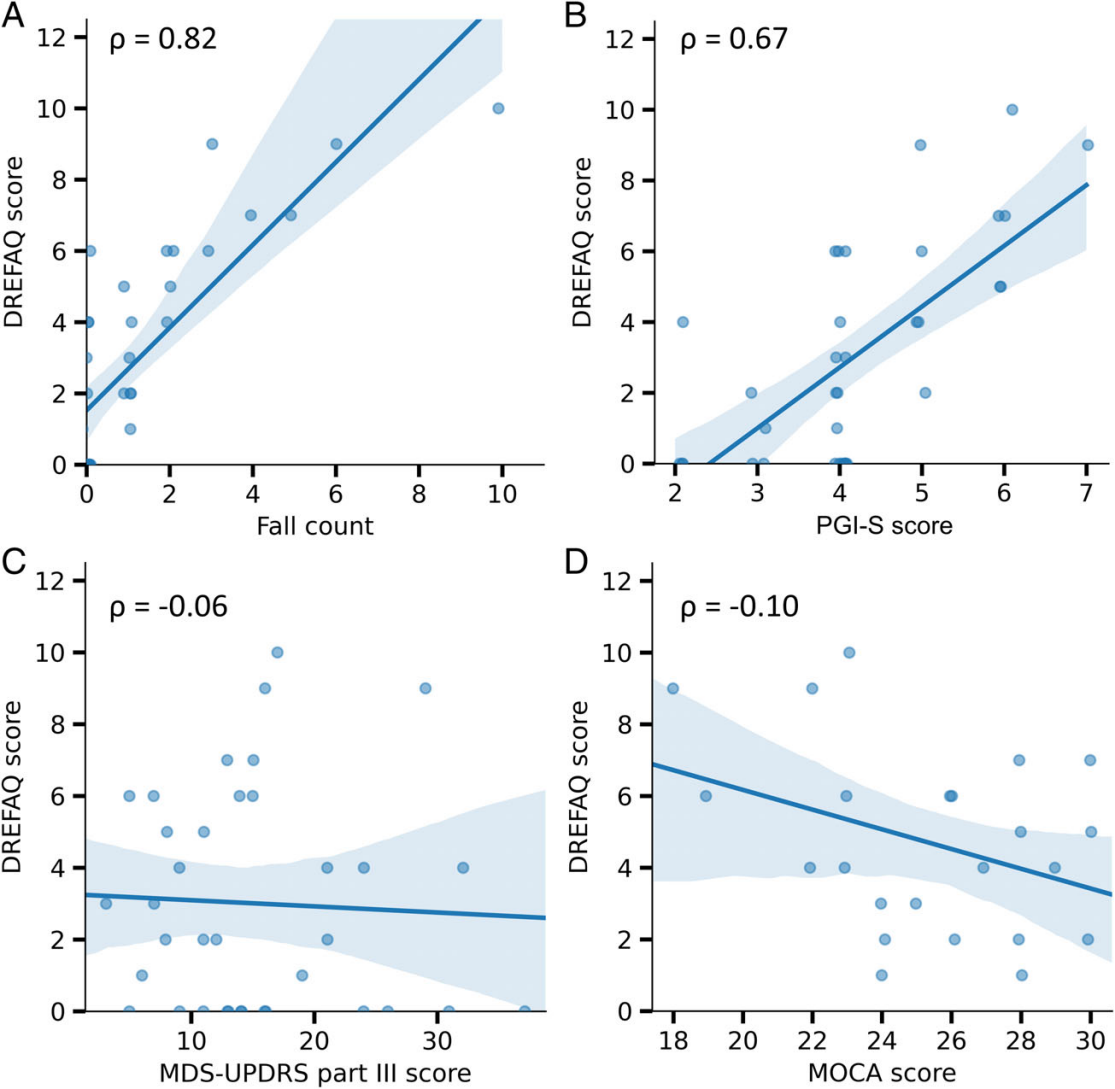
Correlations of the DREFAQ score. (A) The DREFAQ shows a strong correlation with the gold standard for fall documentation (fall count from diary; Spearman's ρ = 0.82; *P* < 0.0001). (B) The highest correlation for other clinical scales was found on the PGI‐S (Spearman's ρ = 0.67; *P* < 0.0001). (C and D) No correlation, however, was found between the MDS‐UPDRS Part III total score (Spearman's ρ = −0.06; *P* = 0.7235) and the MoCA (Spearman's ρ = −0.10; *P* = 0.5805). DREFAQ, Dresden Fall Questionnaire; MDS‐UPDRS, Movement Disorder Society–Sponsored Revision of the Unified Parkinson's Disease Rating Scale; MoCA, Montreal Cognitive Assessment; PGI‐S, Patient Global Impression of Severity.

The DREFAQ score showed a significant positive correlation with patient‐reported falls (Spearman's ρ = 0.82; *P* < 0.0001; see Fig. 1A) and near falls (Spearman's ρ = 0.78; *P* < 0.0001). The correlation of the first item (“How often did you fall?”) with patient‐reported falls was very strongly positive (Spearman's ρ = 0.95; *P* < 0.0001). Similarly, the correlation between the second item (“How often have you been in situations where you almost fell but were able to catch yourself (near falls)?”) and patient‐reported near falls was strongly positive (Spearman's ρ = 0.79; *P* < 0.0001).

To further validate the questionnaire, patients were classified as “fallers” if at least 1 fall was documented in the fall diary and “nonfallers” if no fall was documented. The sensitivity of item 1 of the DREFAQ to detect a faller was 100%, and the specificity to detect nonfallers was 94.7%. An equivalent analysis was performed with near falls and item 2 of the DREFAQ. Item 2 showed a 100% sensitivity to detect near fallers and an 84% specificity to detect non‐near fallers. Of the patients, 2/36 overestimated the number of falls (item 1) and 4/36 overestimated the number of near falls (item 2) in the past 3 months. Interestingly, no patient underestimated the true number of falls or near falls.

The concurrent validity of items 4 (injuries/locations) and 5 (circumstances) was estimated based on the report forms for injuries and circumstances related to the fall. As only 11/18 patients with at least 1 fall event returned the correct number of report forms according to the fall diary, all other patients were excluded from the analysis. All 11 patients recorded their most severe injuries and locations of these injuries correctly in item 4 (type of injury and location). For circumstances of falls (item 5), the most frequently reported circumstance from the report forms was used as the gold standard measure. The circumstances of falls were reported correctly in 10/11 patients.

To further investigate the validity, the DREFAQ total score was compared (Spearman's ρ) with the established clinical characteristics known to affect the risk of calls in PwPD (Table 3). Moderate to low correlations in the expected direction were found for the majority of measures. Unexpectedly, there was only a negligible correlation of falls with age, with the extent of PD motor symptoms as reported by the MDS‐UPDRS Part III and with cognitive performance as reported by MoCA scores. The comparison of DREFAQ fallers (item 1: values >0) versus DREFAQ nonfallers (item 1: values = 0) showed similar results as the correlation analysis (Table 3). These findings highlight the notion that falls are an important dimension of PD symptoms that is not captured well by current scales.

**TABLE 3:** Correlations with other instruments and comparison of fallers and nonfallers

Clinical Variables	Correlation Coefficient to DREFAQ Total Score (Spearman's ρ)	Faller, Item 1 > 0; Median (25th–75th Percentiles) or Mean (SD)	Nonfaller, Item 1 = 0; Median (25th–75th Percentiles) or Mean (SD)	*P* Value: Faller Versus Nonfaller, MWU Test
PGI‐S score	0.67	5 (4–6)	4 (3–4)	**0.0003**
FOG‐Q score	0.51	8.9 (5.18)	6.4 (6.94)	**0.0365**
PAS score	0.50	8.7 (10.97)	4.7 (7.53)	**0.0429**
H&Y stage	0.51	2 (2–3)	3 (2.25–3)	**0.0217**
Disease duration	0.48	14.4 (8.05)	8.9 (6.52)	**0.0117**
FAQ score	0.46	4.6 (3.29)	2.9 (3.00)	**0.0451**
PDQ‐8 score	0.43	15.8 (5.59)	12.1 (4.80)	**0.0152**
BDI‐II score	0.43	12.1 (8.06)	6.3 (6.87)	**0.0284**
PIGD score	0.41	0.9 (0.60)	0.7 (0.66)	0.1161
FES‐I	0.38	34.5 (12.09)	26.9 (12.05)	**0.0205**
BIS‐11 attention subscore	0.36	17.7 (3.46)	14.8 (3.39)	**0.0085**
BIS‐11 score	0.18	62.4 (14.76)	55.6 (10.92)	0.0890
Age	0.12	63.4 (12.57)	67.5 (10.37)	0.2051
SCOPA‐AUT score	0.11	5.0 (3.61)	5.9 (4.62)	0.2783
MDS‐UPDRS Part III	−0.06	14.6 (8.04)	16.1 (8.66)	0.3230
MoCA score	−0.10	25.3 (3.59)	25.4 (3.05)	0.4809

Data are from the validation cohort. Significant differences between groups are highlighted in bold. For the SCOPA‐AUT, only items 8 to 16 were summed. Abbreviations: DREFAQ, Dresden Fall Questionnaire; SD, standard deviation; MWU, Mann–Whitney *U*; PGI‐S, Patient Global Impression of Severity; FOG‐Q, Freezing of Gait Questionnaire; PAS, Parkinson Anxiety Scale; H&Y, Hoehn & Yahr; FAQ, Functional Activities Questionnaire; PDQ‐8, Parkinson's Disease Questionnaire–8; BDI‐II, Beck Depression Inventory–II; PIGD, postural instability and gait disorder; FES‐I, Falls Efficacy Scale–International; BIS‐11, Barratt Impulsiveness Scale–11; SCOPA‐AUT, Scales for Outcomes in Parkinson's Disease–Autonomic Dysfunction; MDS‐UPDRS, Movement Disorder Society–Sponsored Revision of the Unified Parkinson's Disease Rating Scale; MoCA, Montreal Cognitive Assessment.

To identify possible predictors of falling and injury in the validation cohort, we fitted linear models with the number of falls and DREFAQ item 4 as independent variables. For the number of falls, the best model contained age, disease duration, anxiety (PAS score), and impulsivity (BIS‐11 score), with an adjusted *R* ^2^ = 0.737 and *P* < 0.0001. The best predictor for injuries was the PGI‐S severity subscore, which showed an adjusted *R* ^2^ of 0.441. This tight association confirms that fall‐related injuries have an important impact on patients' well‐being.

## Discussion

In this study, we developed a comprehensive questionnaire to evaluate falls, near falls, fear of falling, fall‐related injuries, and circumstances in which falls occur. It was tested in 2 cohorts of PwPD and can be used both in clinical practice and research.

The DREFAQ includes a quantitative part focusing on the incidence of falls, near falls, fear of falling, and severity of fall‐related injuries (4 items on a 4‐point ordinal scale) as well as a qualitative part supplying the clinician with additional information about the location of the fall‐related injuries and information pointing toward different causes of falls. Such observations might contribute to the identification of key impairments amenable to intervention and hence guide the prescription of evidence‐based treatments. The DREFAQ showed high internal consistency with a Cronbach's α of 0.84 (95% confidence interval, 0.73–0.91) and good to excellent convergent validity in comparison with the gold standard (fall diary; Spearman's ρ = 0.77–0.95).

To our knowledge, there was no fall‐specific questionnaire for PwPD when we initiated the study. During the preparation of this article, a group from Australia developed such a questionnaire, the PD‐Specific Falls Questionnaire (PDF‐Q).^44^ There are several differences between the PDF‐Q and the DREFAQ. The PDF‐Q has not been validated, and the recall length of 12 months was considered too long by the authors, which is represented by a high percentage of missing answers. In addition, the PDF‐Q is quite extensive (6 domains with 37 questions in total, including 15 matrix questions and free‐text answers), potentially limiting its use in the daily routine and possibly leading to respondent fatigue, especially in trials with multiple questionnaires.^45^

The incidence of falls in our cohorts was even higher than the numbers published (50% within 3 months), underlining the relevance of this complication.^1^ The personal and economic impacts of falls were highlighted by the 4 fractures in 82 patients in a 3‐month interval. The incidence of severe injuries was higher than in comparative prospective studies^1^, ^46^ or larger population‐based analyses.^47^ This could be associated with the relatively advanced Hoehn & Yahr stages in the confirmation cohort.

In our cohort, correlations between global clinical measures of disease severity with the DREFAQ scores were relatively modest (MDS‐UPDRS Part III total score and MoCA). This highlights the importance of using a fall‐specific assessment tool. Moderate correlations were found on scales evaluating specific motor abilities (FOG‐Q and PIGD score) and scales representing disease severity with functional disability (Hoehn & Yahr stage and FAQ). Consistent with the high personal impact of falls, higher DREFAQ scores were associated with higher self‐perceived disease severity (PGI‐S), but also higher scores for depression and anxiety and lower scores for quality of life (BDI‐II, PAS, and PDQ‐8). These findings are consistent with previous findings by others.^3^, ^4^

EFA and CFA revealed a 2‐factor structure composed of “severe falls with injuries” and “near falls with fear of falling” in both cohorts despite significant demographic differences between the cohorts. Accordingly, fear of falling has been demonstrated to be influenced by factors other than falls (eg, nonmotor symptom burden, cognitive disorders)^48^ and to be a better predictor of quality of life and patients' perceived severity of disease than actual falls.^49^ Fear of falling is also associated with avoidance behavior and might result in overcautious restriction of mobility^50^, ^51^ making it an important target for intervention.^15^, ^52^, ^53^

Self‐rated impulsivity, specifically the attentional domain (BIS‐11 and BIS‐11 attention subscale), was a predictor of falls, which aligns with a previous study.^54^ Attentional impulsivity results in a higher level of distractibility, which can likely influence gait and postural control in PwPD.^29^ Another study demonstrated that patients with the PIGD subtype tended to make more impulsive errors in computerized tests than tremor‐dominant patients, which indicated that motor impulsivity (the inability to control prepotent, impulsive actions) correlated with higher fall risk.^55^ Impulsivity, too, can be addressed by medication changes and therefore constitutes a target for interventions.

The limitations of our study include the reliance on a single center for the validation cohort and a relatively small sample, which did not allow us to reliably determine predictors for recurrent fallers. Such an analysis could be helpful to better identify patients with a specifically negative prognosis, but it is beyond the scope of a scale validation study and will be addressed in a larger population of patients. Further analyses will also include responsiveness to evaluate whether the DREFAQ can detect improvement after therapeutic interventions.

In conclusion, the DREFAQ questionnaire has good reliability, validity, specificity, and sensitivity and is a short instrument for assessing falls in PwPD. A combination of clinical findings and the DREFAQ may assist in routine clinical practice to identify patients with recurrent falls, hint toward specific causes, and help find physiotherapeutic or pharmacological interventions for patients to prevent falls.

## Author Roles

(1) Research Project: A. Conception, B. Organization, C. Execution; (2) Statistical Analysis: A. Design, B. Execution, C. Review and Critique; (3) Manuscript Preparation: A. Writing of the First Draft, B. Review and Critique; (4) Responsibility: A. Integrity of the Data, B. Accuracy of the Data Analysis.

A.F.: 1A, 1B, 1C, 2A, 2B, 3A, 4A, 4B

J.B.: 1A, 1B, 1C, 2A, 2B, 3A, 4A, 4B

S.F.: 1B, 1C, 3B

T.H.: 1B, 1C, 2B, 3B

N.S.: 1C, 3B

T.F.: 1C, 3B

H.R.: 3B

B.H.F.: 1A, 1B, 2A, 2C, 3A, 3B, 4A, 4B

## Disclosures

**Ethical Compliance Statement:** We confirm that we have read the journal's position on issues involved in ethical publication and affirm that this work is consistent with those guidelines. Written informed consent was obtained from all participants before inclusion. All procedures were performed following relevant guidelines and regulations. The study was approved by the institutional review board of the Technische Universität Dresden (BO‐EK‐14012021 and BO‐EK‐517112020).

**Funding Sources and Conflicts of Interest:** The authors report no potential conflict of interest related to the research in this article. This research did not receive any specific grant from funding agencies in the public, commercial, or not‐for‐profit sectors.

**Financial Disclosures for the Previous 12 Months:** A.F. received speaker's honoraria from Zambon and Bial. J.B. received speaker's honoraria from Desitin and Bial. B.H.F. has received honoraria for consultancy from Bial, Desitin, Stadapharm, UCB, and Zambon and grants from Deutsche Forschungsgemeinschaft, The International Parkinson and Movement Disorder Society, and Sächsisches Staatsministerium für Soziales und Gesellschaft, Bundesministerium für Gesundheit. H.R. has received honoraria for consultancy from Bial, Desitin, Kyowa Kirin, Stadapharm, UCB, and Zambon. All other authors declare that there are no additional disclosures to report.

## Supporting information


**Table S1.** Results of the Dresden Fall Questionnaire (DREFAQ).

**Table S2.** DREFAQ German English.
