# Supplementary material for: Development and Validation of a Fall Questionnaire for Patients with Parkinson's Disease
Source: Mov Disord Clin Pract. 2022 Jul 23;9(7):900–8. doi: 10.1002/mdc3.13515 (PMC9547135; doi:10.1002/mdc3.13515)
Supplement: Supplementary file 1 — Table S1. Results of the Dresden Fall Questionnaire (DREFAQ). [file MDC3-9-900-s002.pdf]

Supplementary Material

„Development and Validation of a Fall Questionnaire for Patients with Parkinson's Disease"

Table S1: Results of the DREFAQ

	Validation cohort	Confirmation cohort (baseline)	Confirmation cohort (3 months)
**Falls (n)**			
• Not at all	18	23	27
• 1-2 times in the last 3 months	12	15	11
• 1-4 times per month	5	7	4
• At least 1x per week	1	2	5
**Near falls (n)**			
• Not at all	16	16	13
• 1-2 times in the last 3 months	8	14	17
• 1-4 times per month	7	7	10
• At least 1x per week	5	10	7
**Fear of falling (n)**			
• Not at all	24	12	10
• 1-2 times in the last 3 months	5	11	10
• 1-4 times per month	4	10	12
• At least 1x per week	3	14	14
**Injuries from falls or near falls (n)**			
• Not at all	5	3	3
• Bruises/scratches	16	17	14
• Laceration/cut	3	2	4
• Broken bone	1	5	3
• Other	1	1	1
**Locations of injury, multiple possible (n)**			
• Head	2	6	7
• Trunk	2	8	7
• Shoulder/Arm/Hand	9	14	10
• Hip/Leg/Foot	14	23	14
**Situations of falls (n)**			
• Stumbling fall	8	11	7
• Fall in the dark	1	1	2
• Lack of Balance	14	13	6
• Freezing of Gait	3	7	7
• Lightheadedness/Fainting	1	3	1
• Other	2	3	4

The table shows the total number of answers (n) for each item of the DREFAQ in the validation cohort and the confirmation cohort (baseline; 3 months).
