# Supplementary material for: Development and Validation of a Fall Questionnaire for Patients with Parkinson's Disease
Source: Mov Disord Clin Pract. 2022 Jul 23;9(7):900–8. doi: 10.1002/mdc3.13515 (PMC9547135; doi:10.1002/mdc3.13515)
Supplement: Supplementary file 2 — Table S2. DREFAQ German English. [file MDC3-9-900-s001.pdf]

Dresdner Sturz-Fragebogen

Stürze können Menschen während ihres gesamten Lebens ereilen und aus unterschiedlichen Gründen auftreten. Gesundheitliche Bedeutung haben sie wegen des Risikos von Verletzungen und anderen ungünstigen Folgen für das körperlich-psychische Wohlbefinden.

Erklärung der Begriffe:

Stürze sind Ereignisse, bei denen die betreffende Person unbeabsichtigt auf dem Boden oder einer anderen niedrigeren Fläche aufkommt.

Bei **Beinahe-Stürzen** wird der Sturz durch haltungsstabilisierende Maßnahmen (z.B. Ausfallschritte, Abstützen oder Festhalten) vermieden.

Bitte beantworten Sie die untenstehenden Fragen über Stürze und damit einhergehende Ereignisse ***bezogen auf die letzten 3 Monate.***	**Gar nicht**	**1-2 x in den letzten 3 Monaten**	**1-4 x pro Monat**	**Mindestens 1 x pro Woche**
**1.) Wie oft sind Sie gestürzt bzw. hingefallen?**	☐	☐	☐	☐
**2.) Wie oft ist es zu Situationen gekommen, in denen Sie beinahe gestürzt oder hingefallen sind, sich aber noch abfangen konnten?**	☐	☐	☐	☐
**3.) Wie oft hatten Sie Bedenken oder Angst, zu stürzen bzw. hinzufallen?**	☐	☐	☐	☐

4.) Haben Sie sich bei den Stürzen verletzt? Falls Sie sich verletzt haben, geben Sie bitte Art und Ort Ihrer Verletzung(en) an:

☐ Gar nicht

☐ Prellung/ blauer Fleck/ Schürfwunde

□ Kopf □ Rumpf □ Schulter/ Arm/ Hand □ Hüfte/ Bein/ Fuß

☐ Platzwunde / Schnittwunde (durch Sturz auf spitzen Gegenstand)

□ Kopf □ Rumpf □ Schulter/ Arm/ Hand □ Hüfte/ Bein/ Fuß

☐ Knochenbruch

□ Kopf □ Wirbel/Rippen □ Schulter/ Arm/ Hand □ Hüfte/ Bein/ Fuß

☐ Sonstige: ____

5.) Falls Sie gestürzt sind, geben Sie bitte die Umstände an:

☐ Stolpersturz (z.B. unebene Fläche, Treppenstufen, Teppichkanten)

☐ Sturz im Dunkeln (z.B. beim nächtlichen Toilettengang)

☐ Fehlendes Gleichgewicht (z.B. bei Drehungen im Gehen, vornehmlich Stürze nach hinten)

☐ Festfrieren beim Gehen oder Loslaufen („Freezing“, vornehmlich Stürze nach vorn)

☐ Schwarzwerden vor den Augen oder Bewusstseinsverlust (z.B. nach dem Aufstehen)

☐ Sonstige: ____

Vielen Dank für Ihre Teilnahme!

Anleitung zur Auswertung:

Der Dresdner Sturz-Fragebogen (DREFAQ) ist ein schnell durchführbares Instrument für das Screening und Monitoring von Stürzen, Beinahe-Stürzen und Sturzfolgen bei der Parkinsonerkrankung. Der Fragebogen ist vom Patienten selbst auszufüllen. Die Zeitdauer für das Ausfüllen beträgt ca. 5 Minuten.

Die Fragen 1 bis 4 werden mit 0 bis 3 Punkten bewertet. Falls bei Frage 4 eine „sonstige“ Verletzung genannt wird, schätzen Sie bitte den Schweregrad ein: 1 = leicht, 2 = mittel, 3 = schwer.

Die Punktzahlen aus den Fragen werden zu einem Gesamtscore summiert (insgesamt 0-12 Punkte).

Die Sturzfrequenz ergibt sich direkt über die Frage 1.

Frage 5 gibt eine qualitative Auskunft über die Sturzumstände und damit mögliche Ursachen (eher umweltbedingt, neuropathisch, posturale Instabilität bzw. Freezing).

Gesamtscore: __________________

Sturzfrequenz: _________________

Sturzumstände: _________________

Dresden Fall Questionnaire

Falls can happen throughout life and occur for a variety of reasons. They have health implications because of the risk of injuries and other unfavorable consequences for physical and psychological well-being.

Explanation of terms:

Falls are events in which the individual unintendedly hits the ground or other lower surfaces.

In the case of **near-falls,** the fall is avoided by measures that stabilize posture (e.g. big steps, arm support, or holding onto something).

*Please answer the questions below about falls and related events **based on the previous 3 months.***	**Not at all**	**1-2 times in the previous 3 months**	**1-4 times per month**	**At least once per week**
**1.) How often did you fall?**	☐	☐	☐	☐
**2.) How often have you been in situations where you almost fell but were able to catch yourself (near-falls)?**	☐	☐	☐	☐
**3.) How often did you worry about or fear falling?**	☐	☐	☐	☐

4.) Did you injure yourself during falls? If you have been injured, please specify the type and location of your injury/injuries:

☐ Not at all

☐ Bruises/scratches

□ Head □ Trunk □ Shoulder/ Arm/ Hand □ Hip/leg/foot

☐ Laceration/cut (due to fall on pointed object)

□ Head □ Trunk □ Shoulder/ Arm/ Hand □ Hip/leg/foot

☐ Bone fracture

□ Head □ Vertebrae/ribs □ Shoulder/ Arm/ Hand □ Hip/leg/foot

☐ Other: ____

5.) If you have fallen, please specify the circumstances:

☐ Tripping (e.g. uneven surface, steps, carpet edges)

☐ Fall in the dark (e.g. when going to the bathroom at night)

☐ Lack of balance, e.g. when turning while walking (primarily falls towards the back)

☐ Glued to the ground while walking or initiating a movement ("freezing", mainly falls forward)

☐ Lightheadedness or fainting, e.g., after standing up.

☐ Other: ____

Thank you for your participation!

Instructions for evaluation:

The Dresden Fall Questionnaire (DREFAQ) is a quick-to-perform instrument for screening and monitoring falls, near-falls, and fall consequences in Parkinson's disease. The questionnaire is to be filled out by the patient. The time required for completion is approx. 5 minutes.

Questions 1 to 4 are rated with 0 to 3 points. For question 4: If an injury is entered into "other", please rate the severity as 1 = mild, 2 = moderate, 3 = severe.

The scores from all four questions are summed to give a total score (0-12 points in total).

The fall frequency results directly from question 1.

Question 5 provides qualitative information about the circumstances of the fall and possible causes (e.g. environment, neuropathy, postural instability, freezing).

Total score: _________________

Fall frequency: _________________

Fall circumstances: _________________
